# [Corrigendum] CDDO, a PPAR-γ ligand, inhibits TPA-induced cell migration and invasion through a PPAR-γ-independent mechanism

**DOI:** 10.3892/ol.2026.15615

**Published:** 2026-04-24

**Authors:** Hye-Yeon Jang, On-Yu Hong, Hyun Jo Youn, Jaeuk Jung, Eun Yong Chung, Sung Hoo Jung, Jong-Suk Kim

Oncol Lett 24: 354, 2022; DOI: 10.3892/ol.2022.13474

Following the publication of the above paper, it was drawn to the authors’ attention by an interested reader that, regarding the cell invasion assay experiments shown in Fig. 4A on p. 6, the ‘CON’ and ‘CDDO’ data panels were apparently identical, suggesting that this figure had been assembled incorrectly.

The authors have re-examined their original data, and realize that the same representative microscopic image was mistakenly used for both the control (‘CON’) and CDDO-treated (‘CDDO’) groups in the invasion panels of Fig. 4A. The image for the CDDO-treated group in the invasion panel has now been replaced with the correct original image, and the revised version of Fig. 4 is shown on the next page. Note that this correction is limited to the visual representation of this specific panel in Fig. 4A, and does not affect the quantitative data in either Fig. 4B and 4C or the overall scientific conclusions of the article. The authors are grateful to the Editor of *Oncology Letters* for allowing them the opportunity to publish this Corrigendum, and all the authors agree with its publication. They also thank the reader of the article for drawing this matter to their attention.

## Figures and Tables

**Figure 4.**
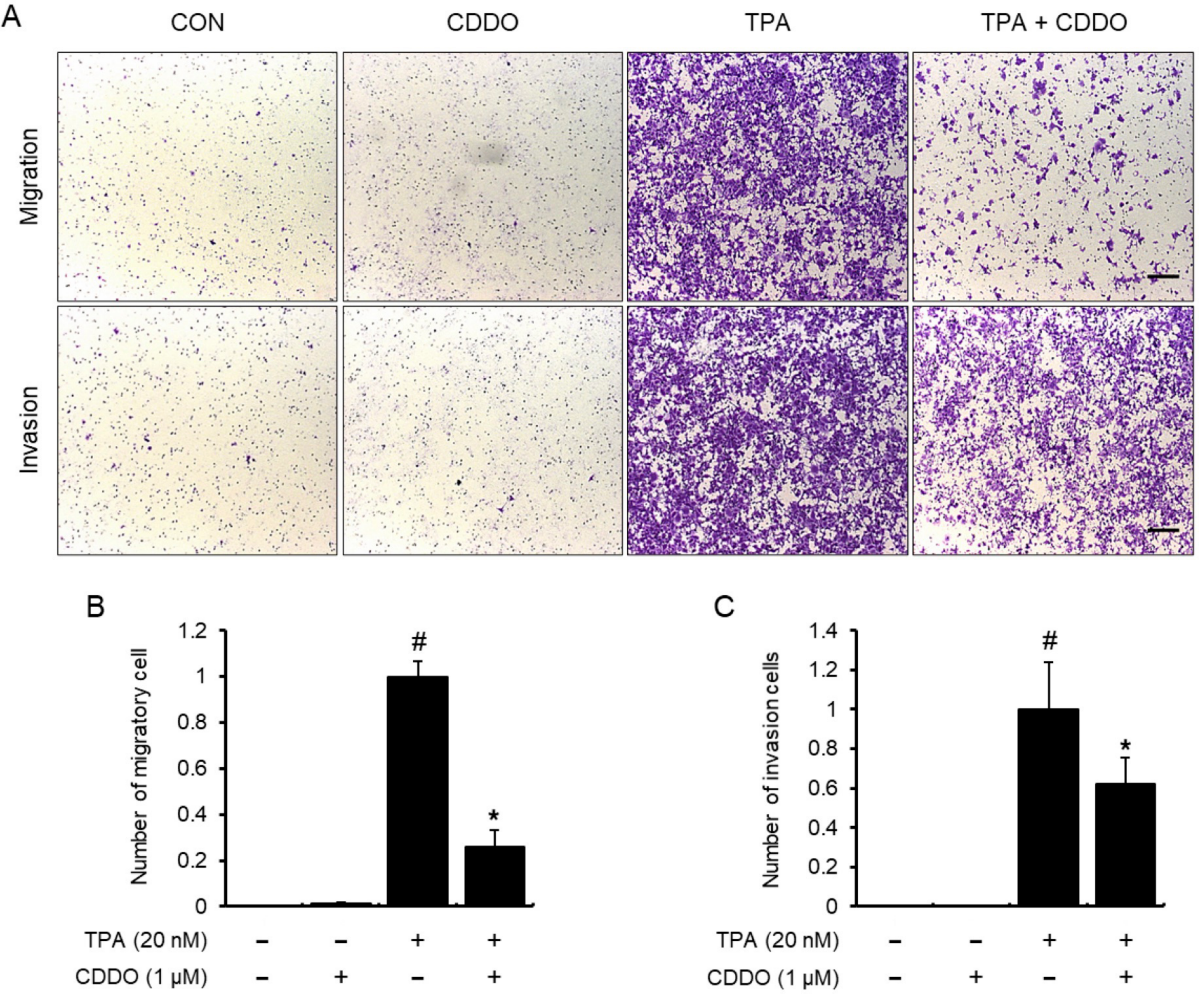
CDDO suppression of migration and invasion of MCF-7 cells. (A) Chamber migration and invasion assays were implemented without or with Matrigel. Cells were treated with CDDO and/or TPA. After 24 h, cells were stained, and microscopic photography was conducted. Bar, 200 µm. (B) Data were quantified by counting the migrated cells in four randomly selected regions during the migration assay. (C) Data were quantified by counting the invading cells in four randomly selected regions during the invasion assay. Values are the mean ± standard error of the mean of three independent experiments. #P<0.05 vs. untreated cells, *P<0.05 vs. TPA alone. CDDO, 2-cyano-3,12-dioxo-oleana-1,9(11)-dien-28-oic acid; TPA, 12-O-tetradecanoylphorbol-13-acetate.

